# The Impact of Atrial Fibrillation on All Heart Chambers Remodeling and Function in Patients with Dilated Cardiomyopathy—A Two- and Three-Dimensional Echocardiography Study

**DOI:** 10.3390/life13061421

**Published:** 2023-06-20

**Authors:** Maria L. Iovănescu, Diana R. Hădăreanu, Despina M. Toader, Cristina Florescu, Octavian Istrătoaie, Ionuţ Donoiu, Constantin Militaru

**Affiliations:** 1 Department of Cardiology, University of Medicine and Pharmacy of Craiova, 200349 Craiova, Romania; 2 Clinical Emergency County Hospital of Craiova, 200642 Craiova, Romania; 3 Filantropia Clinical Hospital, 200516 Craiova, Romania

**Keywords:** cardiomyopathy, arrhythmia, imaging

## Abstract

Atrial fibrillation is frequently seen in patients with dilated cardiomyopathy (DCM), and its presence impacts the function of the heart, with clinical and prognostic consequences. In this prospective single-center study, we aimed to assess the impact of atrial fibrillation on cardiac structure and function, using comprehensive two- and three-dimensional echocardiography. We included 41 patients with DCM and persistent or permanent atrial fibrillation (38 male, age 58.8 ± 11 years), as well as 47 patients with DCM and in sinus rhythm (35 male, age 58 ± 12.5 years). Cardiac chambers and mitral and tricuspid valves’ structure and function were assessed via standard two-dimensional, speckle-tracking, and three-dimensional echocardiography (3DE). Patients with DCM and atrial fibrillation had a more impaired left ventricular global longitudinal strain, higher 3DE left atrial volumes, and reduced function compared to patients in sinus rhythm in the presence of similar left ventricle volumes. Mitral annulus configuration was altered in atrial fibrillation DCM patients. Also, right heart volumes were larger, with more severe atrial and ventricular dysfunction, despite similar estimated pulmonary artery pressures and severity of tricuspid regurgitation. Using advanced echocardiography techniques, we demonstrated that atrial fibrillation induces significant remodeling in all heart chambers.

## 1. Introduction

Dilated cardiomyopathy (DCM) is defined by the presence of left ventricular (LV) or biventricular dilation and systolic dysfunction in the absence of abnormal loading conditions (valvular heart disease, arterial hypertension) or coronary artery disease sufficient to cause global systolic impairment. In some cases, LV dilation can be absent, although overt LV systolic dysfunction is present, which led to the proposal of a new category—hypokinetic non-dilated cardiomyopathy—to avoid diagnostic misinterpretations [1,2,3,4]. The heterogenicity and natural history of the disease is explained by the numerous genetic and non-genetic causal disorders, as well as associated comorbidities. Appropriate diagnosis, etiology-based treatment, if possible, and management of coexisting pathological conditions is of paramount importance, since they impact quality of life, survival, and prognosis. Arrhythmias, whether ventricular or supraventricular, impose a significant burden on DCM patients. Often, atrial fibrillation (AF) complicates disease course, promoting clinical deterioration, with an increase in mortality and morbidity. The pathophysiological processes leading to AF development are extremely complex, and may involve specific disease anomalies or non-specific secondary cardiac chambers structural changes [5,6]. Prognostic implications of AF in DCM patients were reported, though the actual impact of AF on left and right cardiac chamber remodeling in patients with DCM using advanced echocardiography techniques has not been extensively studied [7]. Assessment of myocardial deformation using speckle tracking techniques provides incremental information in different clinical settings, including DCM. The technique was initially developed for LV function assessment, complementary to ejection fraction (EF), but has since been extended and can now be used in the evaluation of both left and right heart chambers, offering reliable functional and prognostic information [8]. The usefulness of 3DE is proven in many areas, such as assessment of LV regional wall motion, graphic views of heart valves, and evaluation of shunts and regurgitant lesions, but mostly in the evaluation of cardiac chambers’ volumes by avoiding geometric assumptions [9].

Thus, the scope of the present study was to (1) evaluate the impact of AF on the geometry and function of both left and right heart chambers using two-dimensional speckle tracking echocardiography (2DSTE) and three-dimensional echocardiography (3DE), a (2) compare the results to those found in patients in sinus rhythm (SR) in this specific clinical setting.

## 2. Materials and Methods

### 2.1. Study Population and Design

The present study was a comparative observational study conducted between 1 October 2021 and 1 July 2022 in an in-hospital setting on DCM patients. These patients were referred to our laboratory for clinically indicated transthoracic echocardiography. Inclusion criteria consisted of confirmed DCM diagnosis, as per current guidelines, being aged over 18 years old, and giving consent to be part of the study. Exclusion criteria were the presence of significant coronary artery disease and/or suboptimal echocardiographic window. The initial cohort consisted of 115 patients, of which 27 were excluded (12 patients due to the presence of significant coronary artery disease, 8 patients who had an inadequate echocardiographic window, and 7 patients with uncontrolled heart rates). The final cohort comprised 88 DCM patients. Patients were divided into two groups based on the presence of SR or persistent/permanent AF (as defined by current guidelines). All patients underwent comprehensive clinically indicated transthoracic 2DSTE and 3DE. Heart rate, blood pressure, height, weight, and body surface area (BSA) were measured in all subjects immediately before the echocardiographic examination. Evaluation was performed when patients were in a compensated state to diminish as much as possible the load-dependency effect on several echocardiographic parameters. Measurements were averaged from three cardiac cycles, in case of SR, or five cardiac cycles, in the presence of AF.

### 2.2. Echocardiography

#### 2.2.1. Two-Dimensional Speckle Tracking Echocardiography

Data were acquired using a commercially available ultrasound system (Vivid E95, GE, Vingmed, Norway). The analysis was performed offline using EchoPAC version 204 software (GE Vingmed Ultrasound).

##### Ventricular Deformation Imaging

**Figure 1:**
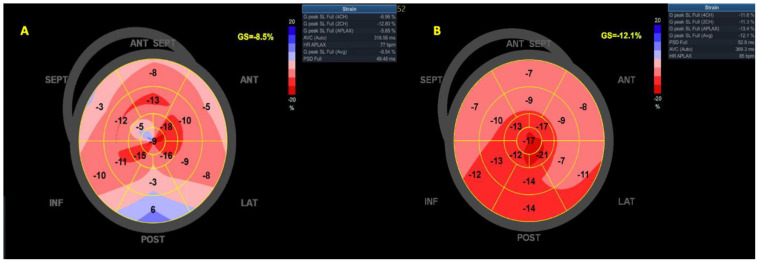
LVGLS with a bull’s eye pattern representation in (**A**). AF—patients or (**B**) SR—patients. Abbreviations: LVGLS—left ventricular global longitudinal strain, SR—sinus rhythm, AF—atrial fibrillation.

For LV global longitudinal strain (LVGLS), a 17-segment model was used. After acquiring the three dedicated apical views (four-chamber, two-chamber, and long-axis views), at a frame rate > 50 fps, endocardial and epicardial borders were tracked. The region of interest (ROI) was carefully defined as not to include the pericardium and, thus, avoid potential reductions in the measurements. Images were generated automatically and manually edited if deemed necessary. Dedicated software was used (AFI LV, EchoPAC v204). End-diastole was selected as the reference point in time and defined based on the peak of the QRS complex [10]. From LVGLS (Figure 1), we also evaluated myocardial work-derived parameters, such as global work index (GWI), i.e., the amount of work performed by the LV during systole; global constructive work (GCW), i.e., positive work performed in systole plus negative work performed in isovolumic relaxation (shortening and lengthening, respectively); global wasted work (GWW), i.e., negative work performed in systole and positive work performed in isovolumic relaxation (lengthening and shortening); and global work efficiency (GWE), i.e., the percentage of constructive work performed over total work (constructive and wasted work) [11].

**Figure 2:**
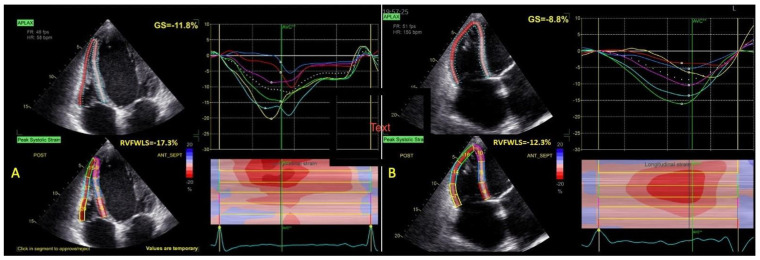
RVFWLS calculation in a patient with (**A**) SR or (**B**) AF. Abbreviations: RVFWLS—right ventricular free wall longitudinal strain; SR, AF—same as in Figure 1.

For the evaluation of right ventricular (RV) strain, the RV-focused apical four-chamber view was obtained, making sure to display the largest basal RV diameter, avoid RV foreshortening, and keep the LV apex at the center of the scanning sector [8,12]. The ROI of the RV was defined in a similar fashion, automatically generated, and manually edited. Although the interventricular septum (IVS) is a contributor to RV systolic performance, since it is mostly a constituent of the LV, RV myocardial deformation was evaluated at the level of the free wall (FW), generating RV free wall longitudinal strain (RVFWLS). The RVFW was divided into three (basal, mid, apical) segments of equal length at end-diastole. Dedicated RV software was used (AFI RV, EchoPAC v204) (Figure 2).

##### Atrial Deformation Imaging

**Figure 3:**
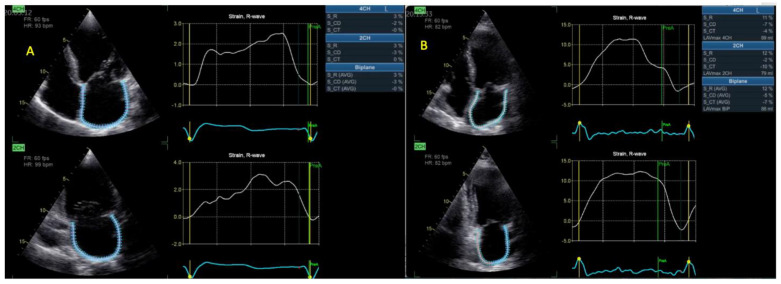
LAS calculation in a patient with (**A**) AF or (**B**) SR. Abbreviations: LAS—left atrial strain; AF, SR—same as in Figure 1.

Left atrial (LA) tracing was performed using dedicated four- and two-chamber views, at adequate gain and depth and with proper orientation, to obtain non-foreshortened images and visualize the entire LA throughout the cardiac cycle [13,14]. Tracing started at the endocardial border of the mitral annulus, progressing up to its opposite side, excluding pulmonary veins and/or LA appendage orifices. A dedicated atrial analysis mode was utilized, i.e., AFI LA, EchoPAC v204. The software automatically identified a 3 mm ROI, which was adjusted to fit the thickness of the LA wall and eliminate pericardial signals. Tracings were compared to the motion of the atrial wall and declined in case of large dropouts. Biplane LA global longitudinal strain (LAS) was calculated in all three phases of LA cycle (reservoir, conduit, and contraction, with the latter option used for patients in sinus rhythm) (Figure 3). Zero-strain reference was set at LV end-diastole [8,15,16]. Tracing the right atrium (RA) started at the lateral tricuspid annulus and ended at the opposite side of the tricuspid annulus, following the endocardial border of the RA lateral wall, roof, and septal wall, in the RV-focused apical four-chamber view. The same principles as used for LA were applied, not only in terms of ROI adjustment, measured strain parameters, and timings, but also regarding satisfactory orientation, gain, and depth.

#### 2.2.2. Three-Dimensional Echocardiography

Complete 2DE and 2DSTE examination was followed by 3DE focused studies using a dedicated probe (M4V, GE, Vivid E95, Vingmed, Asker, Norway). Before 3DE acquisition, 2DE images were optimized. To ensure satisfactory spatial resolution, the pyramidal volumes were optimized to encompass the structures of interest. For a higher temporal resolution, multibeat acquisitions were performed at >20 volumes/second (vps). To avoid stitching artifacts, holding of breath, where possible, was performed by the patients, and acquisitions were undertaken at a relatively stable heart rate. The analysis was conducted offline (EchoPAC v204, GE Vingmed, Asker, Norway).

##### Assessment of the LV

**Figure 4:**
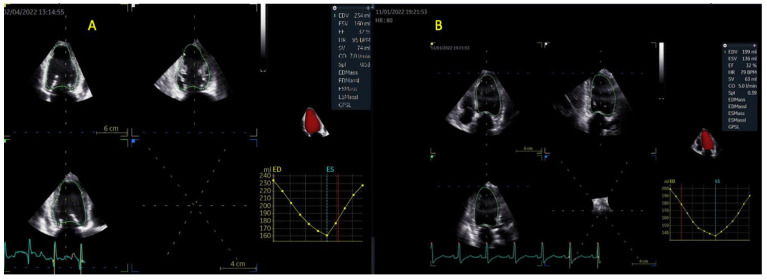
LV volumes and EF calculation via 3DE in a patient with (**A**) AF or (**B**) SR. Abbreviations: LV—left ventricular; EF—ejection fraction; 3DE—three-dimensional echocardiography; SR, AF—same as in Figure 1.

The acquisition of LV full-volume data sets was realized from an apical approach. Off-axis positions were used where considered necessary. To obtain LV volumes and EF, a semi-automated algorithm was utilized (4D autoLVQ, GE HealthCare, Chicago, IL, USA). Firstly, views were correctly aligned. Afterwards, landmarks were set at the level of the base and center of the apex. Selected end-diastolic (ED) and end-systolic (ES) frames were modified, when it was deemed necessary, to ensure proper 3D reconstruction models and waveforms (Figure 4).

##### Assessment of the RV

**Figure 5:**
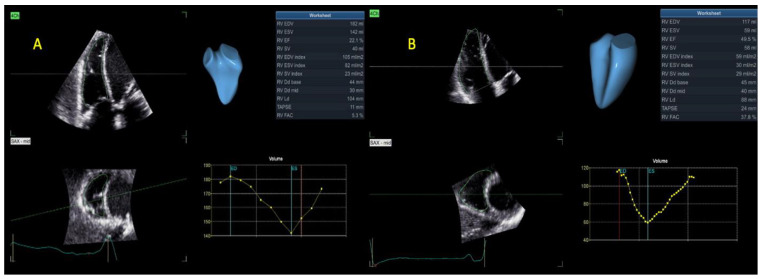
RV volumes and EF calculation via 3DE in a patient with (**A**) AF or (**B**) SR. Abbreviations: RV—right ventricular; EF, 3DE—same as in Figure 4; SR, AF—same as in Figure 1.

Due to its peculiar form, multiple trabeculations, and fibers’ orientation, global assessment of the RV is difficult, with multiple limitations of 2DE that are readily overcome via 3DE evaluation. We used the apical four-chamber view, which was modified to include the entire RV, and acquired full volume data sets at >20 vps. To measure the RV cavity volumes, a semi-automated algorithm was applied (4D autoRVQ). After proper alignment, landmark points were established: two were established at the level of the tricuspid valve; one was established at the RV apex in four-chamber view, the RV/LV anterior, and posterior points; and, lastly, one was established at the RV free wall point in the short axis mid-view. The initial contours were modified manually. The 3D model was visualized and checked in all slices (Figure 5).

##### Assessment of the LA and RA

**Figure 6:**
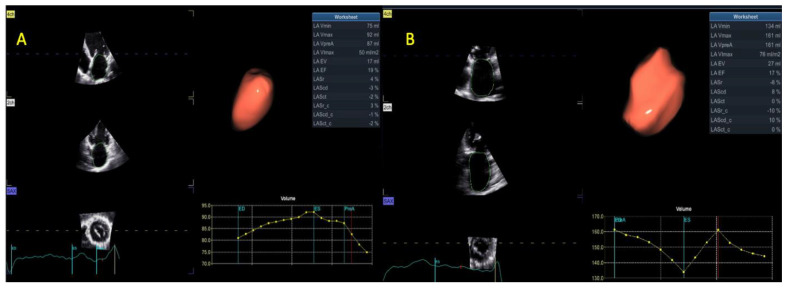
LA volumes and function via 3DE in a patient with (**A**) SR or (**B**). AF; Abbreviations: LA—left atrial; 3DE—same as in Figure 4; SR, AF—same as in Figure 1.

In addition to the strain parameters used to assess LA deformation, 3DE analysis also provides the emptying fraction and an accurate measurement of LA volumes. Care was taken to ensure that complete LA was included. The semi-automated segmentation algorithm used (4D auto LAQ) was initiated by placing one landmark at the mitral valve (MV) at the level of the annulus. The frame was adjusted until the MV was closed. Image position was modified to ensure that the vertical line intersected both the center of the MV and LA roof. Manual editing was performed where circumstances indicated that it was necessary (Figure 6). The same principles were applied to RA 3DE acquisitions.

##### Three-Dimensional Reconstruction of Atrioventricular (Mitral and Tricuspid) Valves

In this context, we also sought to examine the mitral and tricuspid valves with the use of 3DE to obtain a more accurate anatomical and geometrical valvular assessment [17]. To evaluate the morphology of the MV, we acquired full volume data sets, ensuring that the complete valve and annulus were included, at adequate temporal resolution. We used a semi-automated segmentation algorithm (4D autoMVQ). The views were aligned to obtain images where the vertical axis crossed through the center of the MV, and the horizontal axis was parallel to the mitral valve. Six landmarks were placed afterwards: two mitral annulus points, two anterior/posterior points, the coaptation, and the aorta point. Several annulus and leaflets parameters were displayed after checking and adjusting the 3D model. Moreover, 3D TTE acquisitions of the tricuspid valve (TV) were usually performed from an apical approach, using a RV-focused or a foreshortened four-chamber view, to ensure inclusion of the entire TV complex and its anatomical surroundings. The same steps (views alignment, landmarks setting, and manual adjustment where necessary) were followed.

### 2.3. Statistical Analysis

Our analysis was conducted using IBM SPSS Statistics version 28.0.1.1. Baseline characteristics and echocardiographic parameters were defined as mean ± standard deviation (SD) for continuous variables and as absolute number (*n*) or percentage for categorical variables. The normal or skewed distribution of the variables was checked using the Kolmogorov–Smirnov test. To compare data between the two pre-defined groups (AF-group vs. SR-group), we used an unpaired *t*-test. Significance was defined as a two-tailed probability level of <0.05.

## 3. Results

### 3.1. Patients’ Demographics and Clinical Characteristics

The general characteristics of the study population are summarized in Table 1. The SR-group consisted of 47 patients (35 male, age 58 ± 12.5 years), and there were 41 patients in the AF-group (38 male, age 58.8 ± 11 years). Almost 83% of the subjects included were men (74.4% in the SR-group, 92.6% in the AF-group). Age, blood pressure, and heart rate were similar between groups. Regarding the presence of traditional cardiovascular risk factors, dyslipidemia, diabetes mellitus, and systemic hypertension were amongst the most prevalent, with a greater occurrence of dyslipidemia and systemic hypertension in the SR-group (72.3% vs. 63.4%, 38.3% vs. 26.8% respectively). Although approximately one third of the patients in both groups suffered from systemic hypertension, the maximum registered values for the systolic and diastolic blood pressure did not exceed 165 mmHg and 100 mmHg, respectively, thus making arterial hypertension an unlikely cause of ventricular dilation and systolic dysfunction in our group. On the other hand, diabetes mellitus was more prevalent in the AF-group (46.3% vs. 31.9%). However, the differences in the prevalence of cardiovascular risk factors were not statistically significant. Class NYHA II at admission was encountered in 38% patients in the SR-group vs. 8% in the AF-group, along with NYHA III in 38% vs. 26%; 68.3% of the patients in the AF-group were in class NYHA IV at presentation, while only 23.4% of those in SR-group were in the same class (*p* = 0.01) (Table 1).

### 3.2. Conventional 2D, M-Mode and Doppler Measurements

LV-indexed volumes and EF calculated with Simpson biplane method were similar for the two groups (indexed LVEDV 110 ± 26 vs. 117 ± 61 mL/m^2^, indexed LVESV 80 ± 26 vs. 87 ± 52 mL/m^2^, LVEF 29 ± 8 vs. 27 ± 7% in the SR vs. AF group, *p* > 0.05 for all). RV dimensions (basal and mid diameter, length, area) and functional parameters (TAPSE–tricuspid annulus plane systolic excursion, which assesses the longitudinal systolic function of the RV at the level of one segment, and RVFAC–right ventricular fractional area change) had no statistically significant differences between the SR and AF groups. In all patients with at least moderate mitral and/or tricuspid regurgitation, the 2D PISA method was used for quantification of regurgitant lesions. Neither mitral nor tricuspid regurgitant volumes and effective regurgitant orifice areas (EROA) were notably distinct in the two groups (mitral regurgitant volume 39 ± 21 vs. 37 ± 21 mL, MR EROA 28 ± 15 vs. 30 ± 15 mm^2^, tricuspid regurgitant volume 35 ± 25 vs. 44 ± 28 mL, TR EROA 42 ± 32 vs. 50 ± 35 mm^2^ in the SR group vs. the AF group). In both groups, the mean-estimated systolic pulmonary artery pressure (sPAP) was similar (36 ± 19 mmHg vs. 36 ± 18 mmHg, *p* value of 0.9) (Table 2).

### 3.3. 2DSTE Strain Analysis and 3DE Analysis (Volumes, EF, Valvular Assessment)

Similar to 2DE, 3DE LV-indexed volumes and EF were comparable between the two groups (indexed 3DE LVEDV 111 ± 32 vs. 135 ± 68 mL/m^2^, indexed 3DE LVESV 82 ± 25 vs. 96 ± 54 mL/m^2^, 3DE LVEF 31 ± 9 vs. 27 ± 8% in the SR vs. AF group). In the presence of similar LV volumes and EF, LVGLS as an absolute value was significantly lower in the AF group (6.2 ± 2 vs. 7.9 ± 3%, *p* value 0.008), as were myocardial GWI and GCW (474 ± 294 vs. 616 ± 332 mmHg%, 665 ± 365 vs. 822 ± 352 mmHg%, *p* value 0.04), though myocardial GWW and GWE were not (*p* > 0.05) (Table 3). Moreover, 3DE LA maximum and minimum indexed volumes were significantly higher (55 ± 14 vs. 44 ± 13 mL/m^2^, 44 ± 15 vs. 32 ± 14 mL/m^2^), LA function was evaluated via the 3DE LA emptying fraction (LA EF), and biplane left atrium global longitudinal strain in the reservoir phase (LASr) significantly reduced (17 ± 6 vs. 30 ± 15%, 6.9 ± 3 vs. 12.3 ± 7%, respectively) in AF-group compared to patients in SR-group (*p* < 0.05 for all). Unlike in the LV, 3DE RV end-diastolic and end-systolic indexed volumes (RVEDV, RVESV respectively) were significantly larger (67 ± 29 vs. 49 ± 25 mL/m^2^, 43 ± 21 vs. 30 ± 19 mL/m^2^, *p* value 0.01), and the 3DE RV ejection fraction (RVEF) was significantly lower (38 ± 8 vs. 44 ± 10%, *p* value 0.03), in the AF-group compared to the SR-group (Table 4). Similar to the LA, RA 3DE volumes were larger, and the RA function assessed via RA EF and RA global longitudinal strain in the reservoir phase (RASr) were more impaired in the AF-group. Also, patients with AF had a distinct mitral annulus configuration compared to patients in SR, with increased 2D/3D annulus areas, annulus area fractions, and annular diameters. Both MV tenting height and tenting volume were larger in the AF-group. On the other hand, TV geometry was not statistically significantly different between the two groups (Table 5).

## 4. Discussion

Our study demonstrated via the use of advanced transthoracic echocardiographic techniques (i.e., 2DSTE and 3DE) that, in DCM patients, the presence of AF has a significant impact on both left and right heart chambers’ geometry and function. The main findings of our study can be summarized as follows: (i) regarding the left heart, compared to patients with DCM and SR, in the presence of similar 3DE LV volumes, 3DE LVEF, and mitral regurgitation severity grading distribution, patients with DCM and AF exhibited (1) more reduced LVGLS evaluated via 2DSTE, (2) higher 3DE LA maximum and minimum volumes, (3) a more impaired LA function evaluated by 3DE LA emptying fraction and biplane LASr, and (4) an altered mitral annulus configuration (3DE); (ii) regarding the right heart, in the presence of similar estimated pulmonary artery pressures and tricuspid regurgitation severity grading distribution, patients with DCM and AF had (1) higher 3DE RV volumes, (2) a more reduced RV function evaluated via 3DE RVEF and RVFWLS, (3) larger RA maximum and minimum volumes, and (4) a more impaired RA function evaluated via 3DE RA emptying fraction and RASr.

The patients included in our study had similar baseline characteristics in both the AF and SR groups (mean age, heart rate, blood pressure, and prevalence of cardiovascular risk factors), although a more severe clinical presentation was encountered in patients with DCM and AF. After evaluating the left heart chambers and MV, we found that in patients with DCM and AF, compared to those in SR, despite similar 3DE LV volumes, 3DE LVEF and similar distribution of mitral regurgitation severity grading exhibited a significant reduction in LVGLS (absolute values) and myocardial work parameters, such as GWI and GCW. They also displayed increased LA maximum and minimum volumes evaluated via 3DE and reduced LA function evaluated via 3DE left LA emptying fraction (LA EF) and biplane LA global longitudinal strain in the reservoir phase (LASr). In our study, although the presence of AF in DCM patients did not significantly influence the distribution of mitral regurgitation severity grading, it, importantly, modified the configuration of the MV annulus, which was larger. AF was also associated with an increased tethering of the mitral valve (MV). The MV apparatus is an intricate structure, and its optimal functioning mandates a delicate interplay between LV contraction/relaxation, papillary muscles contraction, leaflets, and annular motion. In DCM, functional mitral regurgitation (FMR) occurs due to an imbalance between tethering forces, which are increased (LV global dilation, papillary muscles displacement), and closing forces (reduced LV contractility, asynchronous contraction) in the presence of a structurally normal valve. This imbalance is influenced by the dynamics and dimensions of the mitral annulus (MA) [18,19]. The presence of AF can further modify this complex interplay. On one hand, the larger MA in the AF group could be related to the presence of a more enlarged LA. On the other hand, increased tethering of the MV in the setting of DCM is classically explained based on the presence of global LV remodeling with apical displacement of the papillary muscles, leading to a change in the coaptation point position. Yet, in our study, in the presence of AF, the MV was more tethered, despite comparable LV volumes and function appreciated via LVEF between groups. Patients with DCM-AF presented lower LVGLS and myocardial work indices values. These data suggest that even if LV global remodeling and systolic function (LVEF) are similar, regardless of the presence of AF or SR in DCM subjects, a more reduced LV global longitudinal deformation, possibly caused by the presence of AF, additionally enhances the traction of the MV. One experimental study shows that annular dilation (but not subvalvular LV remodeling) predicts an augmentation in MV tenting volume [20]. In relation to our findings regarding the altered MV annulus geometry in AF-DCM patients, several reports demonstrated that 3DE assessment of mitral annular geometry is able to aid in clinical decision-making regarding MV repair, even in the setting of DCM, and can also predict MR recurrence [21,22].

The fact that AF, as a frequent arrhythmia that often complicates disease course in DCM patients, negatively impacts all heart chambers’ function and geometry in this setting has many implications. In a bicentric study of DCM patients, LVGLS emerged as an independent predictor of adverse outcomes, which exceeded LVEF in optimally treated patients [23]. Various reports associated reduced LA strain with an increased risk of both permanent AF and ischemic stroke occurrence patients with AF [24,25]. Both increased LA volumes and reduced LA function are associated with an impaired prognosis in idiopathic DCM [26].

Regarding the right heart chambers and tricuspid valve, in patients with DCM and AF, compared to patients in SR, although no statistically significant differences in estimated systolic pulmonary artery pressure and tricuspid regurgitation severity grading distribution were noticed between groups, 3DE RV volumes were higher, and RV function evaluated via both 3DE RVEF and RVFWLS was notably reduced. The traditional M-mode and 2DE parameters used in quantifying RV dimensions and function, which were similar between groups, are much less accurate than 3DE. The 3DE RA maximum and minimum volumes were larger, and RA function was also more impaired in AF-DCM patients. The tricuspid valve (TV) in the setting of DCM is mainly, but not exclusively, affected because of secondary pulmonary hypertension, progressive RV dysfunction, and dilation. These changes led, on one hand, to annular dilation mainly in the anterolateral direction due to eccentric forces applied by the RV free wall, and on the other hand to leaflet tethering in the context of papillary muscles displacement. Ventricular functional tricuspid regurgitation (TR) develops as the TV becomes more planar, with reduced excursion, reduced leaflet coaptation, and an increase in tenting volume [27,28,29]. AF occurrence reinforces this vicious circle and can additionally modify this complex interchange. However, in our study, compared to the MV, the TV geometry was not significantly different between groups. Increased RA dimensions and impaired RA function are associated with adverse clinical outcomes in DCM. In a small study, increased RA dimensions predicted an unfavorable response to cardiac resynchronization therapy (CRT) in DCM patients [30]. Finally, the prognostic value of RV dysfunction in the setting of DCM was proven in several studies [31,32].

Although AF is a proven predictor of outcome, to our knowledge, no extensive studies regarding the exact impact on cardiac remodeling and function in DCM of the use of advanced echocardiographic techniques were previously conducted. Our findings might explain more precisely why AF is associated with an impaired prognosis in DCM patients and support the need to follow an extensive echocardiographic multiparametric approach both at baseline and during follow-up when examining DCM patients. After showing the ways that AF impacts the geometry and function of all heart chambers, through our results, we emphasize that, in clinical practice, more intense efforts should be performed to maintain SR in DCM patients. DCM-AF patients should be proposed for AF conversion (whether pharmacological or electrical) or even catheter ablation whenever indicated and considered suitable. Besides the benefits of expanding functional capacity (by increasing cardiac output due to atrial contraction), restoring SR from AF might increase survival in DCM patients.

### Study Limitations

Some limitations can be pointed out. This was a study developed in a single center using a relatively reduced sample of patients. Most subjects included were male, and we cannot ignore the possibility of observing different findings in female patients. Besides exclusion of coronary artery disease, we did not focus on DCM etiology and did not consider the possibility of tachycardia-induced cardiomyopathy, and as such, we were unable to make etiology-based correlations.

## 5. Conclusions

DCM patients with AF showed a more impaired LVGLS evaluated via 2DSTE, higher 3DE LA volumes, and reduced LA function evaluated via 3DE and biplane LASr compared to patients in SR in the presence of similar 3DE LV volumes, 3DE LVEF, and distribution of mitral regurgitation severity grading. Mitral annulus configuration was altered in AF-DCM patients. The 3DE RV volumes were larger and RV function assessed via 3DE RVEF and RVFWLS was more reduced, as were RA volumes and function in AF patients, despite similar estimated pulmonary artery pressures and distribution of tricuspid regurgitation severity grading.

## Figures and Tables

**Table 1:** Patients’ characteristics.

Variable	ALL (*n* = 88)	SR (*n* = 47)	AF (*n* = 41)	*p* Value
Age (y), mean	58 ± 11.8	58 ± 12.5	58.8 ± 11	0.7
Male, *n* (%)	73 (82.9%)	35 (74.4%)	38 (92.6%)	0.02
Body surface area (m^2^)	1.93 ± 0.2	1.89 ± 0.1	1.98 ± 0.2	0.03
NYHA class at admission (II; III; IV), *n*	20; 29; 39	18; 18; 11	2; 11; 28	0.01
Heart rate (beats/min), mean	78 ± 14	76 ± 13	81 ± 14	0.1
Systolic blood pressure (mmHg), mean	113 ± 10	113 ± 10	112 ± 10	0.6
Diastolic blood pressure (mmHg), mean	69 ± 7	69 ± 7	69 ± 7	0.7
Hypertension, *n* (%)	29 (32.9%)	18 (38.3%)	11 (26.8%)	0.5
Diabetes mellitus, *n* (%)	32 (36%)	15 (31.9%)	17 (46.3%)	0.3
Chronic kidney disease, *n* (%)	22 (25%)	9 (19.1%)	13 (31.7%)	0.1
Dyslipidemia, *n* (%)	60 (68.2%)	34 (72.3%)	26 (63.4%)	0.3
Tobacco use, *n* (%)	24 (27%)	11 (23.4%)	13 (31.7%)	0.3
History of ICD implantation, *n* (%)	11 (12.5%)	5 (10.6%)	6 (14.6%)	0.7

Data are presented as number (*n*), percentage (%), and mean ± standard deviation.

**Table 2:** Conventional 2D, M-mode, and Doppler measurements.

Parameter	SR-Group	AF-Group	*p* Value
LVEDV (mL)	206 ± 50	234 ± 115	0.1
LVEDV index (mL/m^2^)	110 ± 26	117 ± 61	0.4
LVESV (mL)	149 ± 48	173 ± 99	0.1
LVESV index (mL/m^2^)	80 ± 26	87 ± 52	0.4
LVSV (mL)	57 ± 13	60 ± 22	0.3
LVEF (%)	29 ± 8	27 ± 7	0.2
RVAd (cm^2^)	21 ± 6	26 ± 11	0.1
RVAs (cm^2^)	14 ± 6	16 ± 8	0.3
RVbasD (mm)	40 ± 8	48 ± 6	0.1
RvmidD (mm)	29 ± 10	36 ± 12	0.3
Rvlength (mm)	74 ± 7	82 ± 35	0.5
TAPSE (mm)	17 ± 3	16 ± 3	0.7
RVFAC (%)	38 ± 11	35 ± 8	0.1
sPAP (mmHg)	36 ± 19	36 ± 18	0.9
TR Rvol (mL)	35 ± 25	44 ± 28	0.4
TR EROA (mm^2^)	42 ± 32	50 ± 35	0.6
MR Rvol (mL)	39 ± 21	37 ± 21	0.8
MR EROA (mm^2^)	28 ± 15	30 ± 15	0.7

Data are presented as mean ± standard deviation. Abbreviations: LVEDV—left ventricular end-diastolic volume, LVESV—left ventricular end-systolic volume, LVESV—left ventricular stroke volume, LVEF—left ventricular ejection fraction, RVAd—right ventricular area in diastole, RVAs—right ventricular area in systole, RvbasD—right ventricular basal diameter, RvmidD—right ventricular medial diameter, Rvlength—right ventricular length, TAPSE—tricuspid annular plane systolic excursion, RVFAC—right ventricular fractional area change, TR Rvol—tricuspid regurgitant volume, TR EROA—tricuspid effective regurgitant orifice area, MR Rvol—mitral regurgitant volume, MR EROA—mitral effective regurgitant orifice area, index—indexed to body surface area.

**Table 3:** All 2D STE analysis results.

Parameter	SR-Group	AF-Group	*p* Value
LVGLS (%)	7.9 ± 3	6.2 ± 2	0.008
BP LASr (%)	12.3 ± 7	6.9 ± 3	<0.001
RVFWLS (%)	17.2 ± 7	13.2 ± 5	0.004
RASr (%)	22 ± 12	9 ± 6	<0.001
PSD (ms)	95 ± 41	95 ± 41	0.8
MYO GWI (mmHg%)	616 ± 332	474 ± 294	0.04
MYO GCW (mmHg%)	822 ± 352	665 ± 365	0.04
MYO GWW (mmHg%)	193 ± 163	144 ± 77	0.08
MYO GWE (%)	77 ± 11	77 ± 9	0.8

Data are presented as mean ± standard deviation. Abbreviations: LVGLS—left ventricular global longitudinal strain, BP LASr—biplane left atrial global longitudinal strain in the reservoir phase, RVFWLS—right ventricular free wall longitudinal strain, RASr—right atrial global longitudinal strain in reservoir phase, PSD—peak strain dispersion, MYO—myocardial, GWI—global work index, GCW—global constructive work, GWW—global wasted work, GWE—global work efficiency.

**Table 4:** All 3DE analysis results.

Parameter	SR-Group	AF-Group	*p* Value
3D LVEDV (mL)	214 ± 56	256 ± 112	>0.05
3D LVEDV index (mL/m^2^)	111 ± 32	135 ± 68	0.08
3D LVESV (mL)	152 ± 51	191 ± 101	>0.05
3D LVESV index (mL/m^2^)	82 ± 25	96 ± 54	0.1
3D LVSV (mL)	60 ± 14	64 ± 21	0.3
3D LVSV index (mL/m^2^)	32 ± 7	31 ± 12	0.6
3D LVEF (%)	31 ± 9	27 ± 8	0.1
3D RVEDV (mL)	93 ± 47	127 ± 59	0.01
3D RVEDV index (mL/m^2^)	49 ± 25	67 ± 29	0.01
3D RVESV (mL)	55 ± 35	80 ± 44	0.01
3D RVESV index (mL/m^2^)	30 ± 19	43 ± 21	0.01
3D RVSV (mL)	38 ± 16	47 ± 19	0.039
3D RVSV index (mL/m^2^)	20 ± 8	25 ± 10	0.058
3D RVEF (%)	44 ± 10	38 ± 8	0.03
3D LAV max (mL)	80 ± 22	107 ± 35	<0.001
3D LAV max index (mL/m^2^)	44 ± 13	55 ± 14	0.002
3D LAV min (mL)	58 ± 25	89 ± 29	<0.001
3D LAV min index (mL/m^3^)	32 ± 14	44 ± 15	0.002
3D LA EF (%)	30 ± 15	17 ± 6	<0.001
3D RAV max (mL)	57 ± 27	87 ± 43	0.001
3D RAV max index (mL/m^2^)	31 ± 14	44 ± 21	0.006
3D RAV min (mL)	40 ± 24	67 ± 35	<0.001
3D RAV min index (mL/m^2^)	22 ± 12	34 ± 19	0.005
3D RA EF (%)	31 ± 12	23 ± 12	0.01

Data are presented as mean ± standard deviation. Abbreviations: LVEDV, LVESV, LVSV, LVEF, index—same as in Table 2. RVEDV—right ventricular end-diastolic volume, RVESV—right ventricular end-systolic volume, RVSV—right ventricular stroke volume, RVEF—right ventricular ejection fraction, LAV max—left atrial maximum volume, LAV min—left atrial minimum volume, LA EF—left atrial emptying fraction, RAV max—right atrial maximum volume, RAV min—right atrial minimum volume, RA EF—right atrial emptying fraction, 3D—three-dimensional.

**Table 5:** All 3DE-derived mitral and tricuspid valve parameters.

Parameter	SR-Group	AF-Group	*p* Value
MV 3D Annulus Area (cm^2^)	12 ± 3	15 ± 3	0.008
MV 2D Annulus Area (cm^2^)	12 ± 3	14 ± 3	0.009
MV Annulus Perimeter (cm)	17 ± 26	14 ± 2	0.4
MV A-P Diameter (cm)	3 ± 0.4	4 ± 0.5	0.003
MV AL-PM Diameter (cm)	3.9 ± 0.5	4.3 ± 0.5	0.02
MV Annulus Height (mm)	5 ± 2	6 ± 2	0.3
MV Saddle Angle (degrees)	159 ± 18	156 ± 16	0.5
MV Annulus Area Fraction (%)	−2 ± 5	0.4 ± 3	0.03
MV Tenting Height (cm)	1 ± 0.3	1.3 ± 0.2	0.002
MV Tenting Volume (mL)	5 ± 2	8 ± 4	0.01
TV 3D Annulus Area (cm^2^)	10 ± 3	12 ± 3	0.053
TV 2D Annulus Area (cm^2^)	10 ± 3	12 ± 2	0.053
TV Annulus Area Change (%)	15 ± 7	13 ± 4	0.2
TV Annulus Perimeter (cm)	11 ± 2	12 ± 2	0.2
TV 4CH Diameter (cm)	3 ± 0.7	4 ± 0.7	0.01
TV Major Diameter (cm)	4 ± 0.7	6 ± 0.8	0.2
TV Minor Diameter (cm)	3 ± 0.6	3 ± 0.7	0.7
TV Tenting Height (cm)	0.7 ± 0.3	0.8 ± 0.2	0.3
TV Tenting Volume (mL)	2 ± 1	3 ± 2	0.1

Data are presented as mean ± standard deviation. Abbreviations: MV—mitral valve, 3D—three-dimensional, 2D—two-dimensional, A-P—antero-posterior, AL-PM—anterolateral-posteromedial, TV—tricuspid valve, 4CH—four-chamber.

## Data Availability

Data are available on request with the approval of the University of Medicine and Pharmacy of Craiova.
